# Recurrent cytarabine-induced sinus bradycardia in a patient with acute myeloid leukemia: a case report and review of the literature

**DOI:** 10.1186/s13256-025-05746-6

**Published:** 2025-12-11

**Authors:** Tsebaot Tesfaye, Elsa Wolde Mamo, Derbe Assefa, Biniyam Barega, Tigist Seleshi, Dejuma Yadeta Goshu

**Affiliations:** 1 Department of Internal Medicine, Addis Ababa University, Addis Ababa, Ethiopia; 2 Addis Ababa University, Addis Ababa, Ethiopia

**Keywords:** Cytarabine, Bradycardia, Acute myeloid leukemia, Cardiotoxicity, Left ventricular systolic dysfunction, Case report

## Abstract

**Background:**

Cytarabine is a pyrimidine nucleoside analog that plays a crucial role in the treatment of acute myeloid leukemia. It is typically used in combination with anthracyclines during both induction and consolidation chemotherapy. The well-known side effects of cytarabine include myelosuppression, mucositis, and gastrointestinal disturbances, while cardiotoxicity is rare. Among the cardiovascular effects, sinus bradycardia is uncommon and often underreported. The exact mechanisms behind this condition remain unclear, but several theories have been proposed. These include direct cytotoxic effects on the cardiac conduction system, autonomic imbalance, metabolic or electrolyte disturbances, and immune-mediated hypersensitivity. Most reported cases of bradycardia are transient and asymptomatic, occurring during or shortly after infusion. Delayed or recurrent instances of bradycardia are particularly uncommon.

**Case presentation:**

We report the case of a 24-year-old Ethiopian female diagnosed with acute myeloid leukemia who developed delayed and recurrent sinus bradycardia accompanied by transient left ventricular systolic dysfunction during cytarabine therapy. Her initial echocardiographic evaluation showed a normal cardiac function, with a left ventricular ejection fraction of 65%. The first episode of asymptomatic bradycardia occurred approximately 1 month after completing a low-dose cytarabine and doxorubicin induction regimen, accompanied by a transient decrease in left ventricular ejection fraction to 50%, which subsequently normalized. During consolidation therapy with intermediate-dose cytarabine, she experienced a second, more pronounced episode of bradycardia. This episode resolved following the temporary discontinuation of cytarabine and close monitoring. Laboratory evaluations ruled out possible causes of bradycardia including electrolyte abnormalities, thyroid dysfunction, and infection. The Naranjo Adverse Drug Reaction Probability Scale yielded a score of 9, indicating a definite drug-related event.

**Conclusion:**

Cytarabine-induced sinus bradycardia is a rare but clinically significant adverse event that may occur even in patients without pre-existing cardiac disease or traditional cardiotoxic risk factors. This case is unique for its delayed onset, recurrence upon rechallenge, and transient decline in left ventricular ejection fraction. Clinicians should maintain vigilance for bradyarrhythmias during both induction and consolidation phases of cytarabine therapy, even at lower doses. Awareness of this phenomenon allows timely recognition, avoidance of unnecessary treatment discontinuation, and safe continuation of potentially curative therapy under appropriate cardiac monitoring.

## Background

Cytarabine, a pyrimidine nucleoside analog, remains a cornerstone of induction and consolidation therapy for acute myeloid leukemia (AML) [1]. While its common toxicities, such as myelosuppression, mucositis, and gastrointestinal disturbances, are well recognized, cardiovascular adverse effects are uncommon [2]. Among these, sinus bradycardia is rare and likely underreported. Available evidence, largely from isolated case reports or small series, typically describes transient, asymptomatic bradycardia occurring during or shortly after cytarabine infusion [3–11]. Consequently, the true incidence, timing, and mechanisms of this phenomenon remain poorly understood.

This case broadens our current understanding of cytarabine-related cardiotoxicity in several important ways. It presents a delayed onset of bradycardia approximately 1 month after completion of a low-dose cytarabine regimen, with recurrence upon rechallenge during intermediate-dose therapy, a pattern seldom described. The patient also developed transient left ventricular systolic dysfunction, possibly influenced by bradycardia-related hemodynamic changes, underscoring the interplay between rhythm abnormalities and ventricular performance. Notably, these events occurred in the absence of pre-existing cardiac disease or conventional risk factors, highlighting the need for vigilance even during lower-dose or consolidation phases of treatment.

By integrating a review of prior cases, this report provides a detailed clinical and contextual analysis of cytarabine-induced bradycardia, reinforcing the importance of cardiac monitoring and multidisciplinary management. It adds to the limited but growing body of literature characterizing the spectrum of cytarabine-induced cardiotoxicity and supports including bradyarrhythmias in the differential diagnosis of chemotherapy-related cardiac events.

## Case presentation

A 24-year-old Ethiopian female presented with a 1-month history of easy fatigability. Associated with that, she had intermittent spontaneous gum bleeding and nasal bleeding for 2 days. She was previously healthy, with no known history of cardiovascular disease, thyroid abnormalities, or other chronic illnesses. She had no prior hospitalizations or surgeries. There was no family history of hematological malignancy, congenital heart disease, arrhythmias, or sudden cardiac death. She was not taking any regular medications before presentation and reported no history of smoking, alcohol, or recreational drug use. She had no known drug allergies. Baseline infectious disease screening, including human immunodeficiency virus (HIV), hepatitis B, and hepatitis C, was negative. She reported no history of syncope, chest pain, palpitations, or exercise intolerance before diagnosis.

On examination, she was pale with conjunctival pallor. Vital signs were normal with a heart rate of 93 bpm, blood pressure of 100/70 mmHg, respiratory rate of 18 breaths per minute, and temperature of 36.7 °C. Cardiac, respiratory, and abdominal examinations were unremarkable. Laboratory investigations showed a white blood cell count of 6.23 × 10^3^/µL with 0% neutrophils, 16.5% lymphocytes, 83.3% monocytes, hemoglobin 8.8 g/dL, hematocrit 24.3%, and platelets 18 × 10^3^/µL. Peripheral smear revealed numerous blasts with irregular nuclei, fine chromatin, and moderate cytoplasm with granules, suggestive of AML. Bone marrow aspirate confirmed AML, and histoimmunocytology supported a diagnosis of acute myeloblastic leukemia. Flow cytometry and cytogenetic studies were not available. Echocardiography demonstrated normal cardiac structure and function with an ejection fraction (EF) of 65%. A baseline electrocardiogram (ECG) was not available. Other baseline laboratory investigations, including renal and liver function, were within normal limits.

The patient was started on induction chemotherapy with the “7 + 3” regimen (cytarabine 100 mg/m^2^/day continuous intravenous (IV) infusion for 7 days and doxorubicin 50 mg/m^2^/day IV infusion over 4 hours for 3 days). Premedications included cimetidine, metoclopramide, ondansetron, and dexamethasone. Five days after completing induction, she developed neutropenic fever of mucosal origin complicated by typhlitis. Blood cultures were obtained and initially showed no growth, and subsequent cultures remained negative. Chest imaging and urine analysis were unremarkable. C-reactive protein (CRP) and procalcitonin were not available. She was treated empirically with cefepime and vancomycin. Due to persistent fever, cefepime was discontinued, and meropenem was added. Liposomal amphotericin B was also initiated for suspected invasive fungal infection, although subsequent chest computed tomography (CT) was normal. During this period, echocardiography revealed that left ventricular systolic function had decreased from a baseline EF of 65% to 50%. Despite antimicrobial therapy, fever persisted until a perianal abscess was identified and surgically drained, after which the fever subsided.

On postoperative day 6, exactly 1 month after she completed the induction chemotherapy, she developed shortness of breath, cough, and easy fatigability, associated with persistent bradycardia with a heart rate of 48 bpm. Electrolytes and thyroid function tests were normal, and infection-related causes were excluded. Chest CT demonstrated pulmonary edema. ECG tracing during this period was not available. She was managed with furosemide and supplemental oxygen for 2 days with complete resolution of respiratory symptoms. Despite possible causes of bradycardia being ruled out, persistent asymptomatic bradycardia with a heart rate of 50 bpm was noted for two additional days before resolving spontaneously to a baseline heart rate of 85 bpm. She was discharged with stable vital signs of blood pressure of 100/70 mmHg, heart rate of 82 bpm, respiratory rate of 20 breaths per minute, temperature of 36.5 °C, and SpO₂ of 94% on room air, on prophylactic ciprofloxacin, fluconazole, and acyclovir. Repeat echocardiography 1 week later showed recovery of systolic function with an EF of 65%.

Two weeks later, consolidation chemotherapy was initiated with intermediate-dose cytarabine (IDAC (Intermediate-Dose-Ara-V); 1.5 g/m^2^ continuous IV infusion over 24 hours on days 1, 3, and 5). During the first cycle of IDAC, she developed neutropenic fever of unknown origin, which responded to 7 days of treatment with vancomycin and meropenem.

**Fig. 1:**
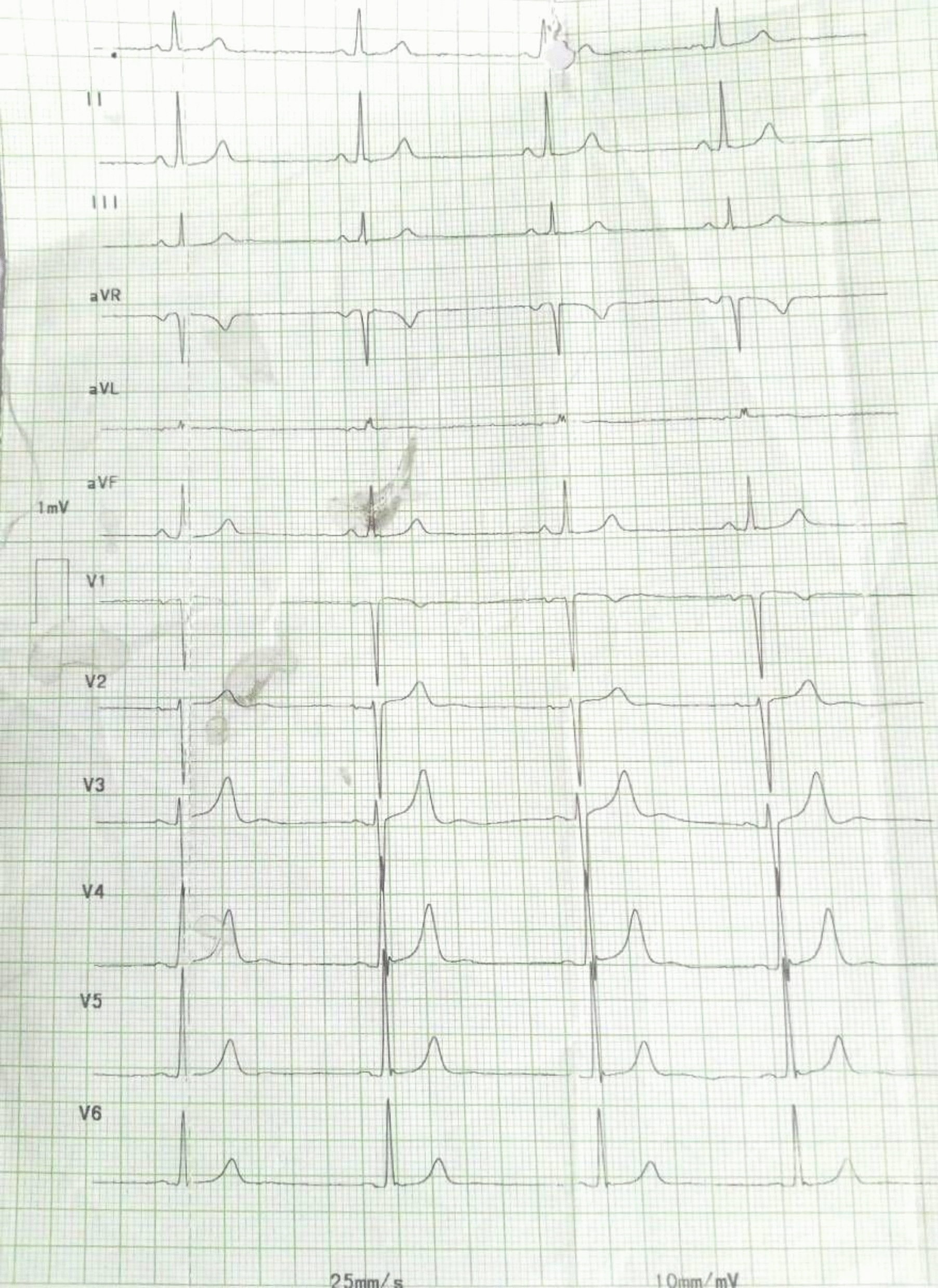
Electrocardiogram (ECG) showing sinus bradycardia (heart rate 51 bpm) during cytarabine infusion in the fourth consolidation cycle

**Fig. 2:**
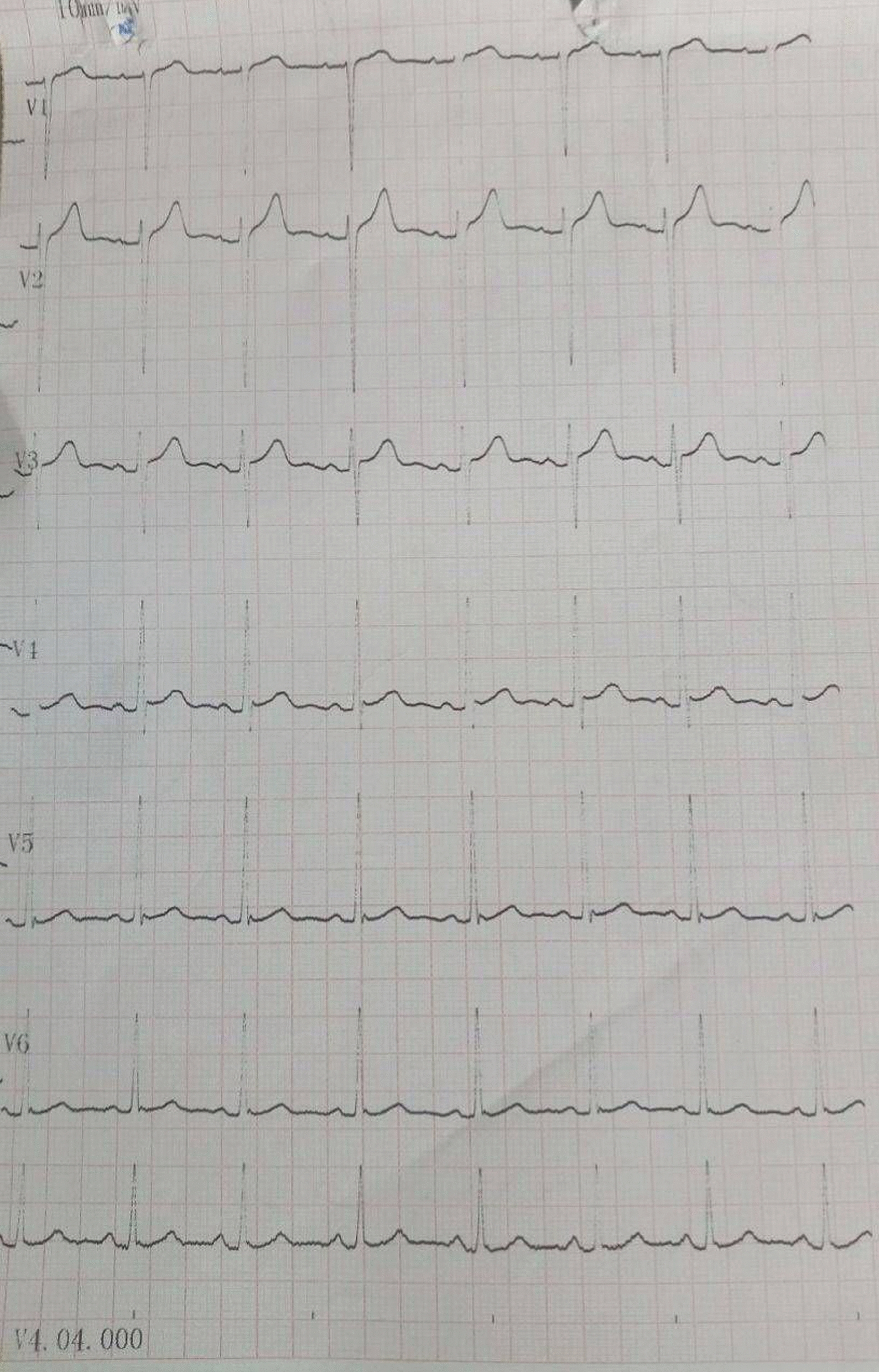
ECG showing resolution of sinus bradycardia with return to normal sinus rhythm (heart rate 93 bpm) after discontinuation of cytarabine

The second and third cycles of IDAC were uneventful. On the second day of the fourth cycle of IDAC, shortly after starting cytarabine infusion, she developed fatigue and dizziness, with sinus bradycardia and with heart rate drop from 80 to 51 bpm (Fig. 1). Electrolytes and thyroid function tests were normal, and other possible causes of bradycardia were ruled out. Cytarabine was temporarily discontinued, her heart rate returned to normal sinus rhythm at 93 bpm, and her symptoms improved without additional intervention (Fig. 2). Five days later, cytarabine was reinitiated under close monitoring, and she completed the cycle without recurrence of bradycardia. Two days after completion, her heart rate remained stable at the baseline heart rate of 85 bpm.

On subsequent follow-ups over one and a half years, the patient remained in remission and clinically stable. She did not require blood transfusions or any subsequent therapy or long-term cardiac follow-up.

## Discussion

Cardiotoxicity is an uncommon but clinically significant complication of cytarabine therapy. While anthracyclines such as doxorubicin are more frequently associated with cardiac adverse effects, cytarabine has also been implicated in various forms of cardiotoxicity, including arrhythmias, pericarditis, myocarditis, and, rarely, sinus bradycardia [12, 13]. Large reviews on chemotherapy-induced cardiotoxicity classify antimetabolites, including cytarabine, as being associated with arrhythmias such as sinus tachycardia and bradycardia; however, the precise incidence of cytarabine-specific bradycardia remains undefined because available data are limited and heterogeneous [12]. Furthermore, bradyarrhythmias related to cytarabine are likely underreported, as they are often transient and asymptomatic, yet may lead to diagnostic confusion or premature discontinuation of therapy.

Cytarabine-induced sinus bradycardia and other conduction abnormalities have been described mainly with intermediate-to high-dose regimens, although isolated cases have occurred at lower doses, particularly in combination with other chemotherapeutic agents [3–11]. Most reports describe symptomatic sinus bradycardia occurring during or shortly after cytarabine infusion, which resolves following drug discontinuation and supportive care, suggesting an acute and reversible process [3–11].

By contrast, our patient developed the first episode of bradycardia approximately 1 month after completing a low-dose cytarabine regimen, accompanied by a transient decline in left ventricular ejection fraction (LVEF) from 65% to 50%, which subsequently normalized. The ECG tracing from this initial episode, as well as a baseline ECG prior to chemotherapy, were not available, limiting the assessment of pre-existing conduction abnormalities. While cytarabine may have contributed, concurrent doxorubicin exposure is a plausible factor given its well-documented, dose-dependent cardiotoxicity. It is also possible that the apparent transient reduction in LVEF was partly due to underestimation of true systolic function secondary to bradycardia, as prolonged diastolic filling and altered preload conditions can artifactually lower the calculated ejection fraction on echocardiography.

The second, more pronounced episode occurred during intermediate-dose cytarabine (IDAC) therapy and resolved following temporary discontinuation and close monitoring. The atypical delayed onset, together with reversible left ventricle (LV) systolic dysfunction, suggests mechanisms extending beyond immediate infusion-related effects and possibly involving synergistic or idiosyncratic interactions between the chemotherapeutic agents.

The patient received standard premedications, including cimetidine, metoclopramide, ondansetron, and dexamethasone. Although rare cases of bradycardia have been reported with cimetidine or rapid IV administration of metoclopramide, such events are extremely uncommon in young, otherwise healthy adults. Ondansetron and dexamethasone are not typically associated with bradycardia. During neutropenic fever, the patient also received antibiotics, including cefepime, meropenem, and vancomycin. While each of these agents has been rarely associated with bradyarrhythmias, the timing of bradycardia relative to these drugs, normal renal function, and absence of electrolyte disturbances make them unlikely contributors. In addition, the patient received liposomal amphotericin B approximately 1 week before the first bradycardia episode; although amphotericin B has been linked to bradyarrhythmias in rare cases, normal electrolytes and the temporal separation make direct causation improbable. Importantly, bradycardia recurred upon cytarabine rechallenge in the absence of all premedications, antibiotics, and antifungal therapy, supporting cytarabine as the primary causative agent of the conduction abnormalities. Nonetheless, these medications remain potential, albeit improbable, confounders that clinicians should consider when evaluating bradyarrhythmias during chemotherapy.

The precise pathophysiology of cytarabine-induced bradycardia remains unclear. Proposed mechanisms include direct cytotoxicity to the sinoatrial or atrioventricular node, autonomic imbalance or reflex vagal activation during infusion, metabolic or electrolyte disturbances, or ischemia unmasking latent conduction abnormalities [5, 7, 14]. More recently, an immune-mediated or hypersensitivity mechanism has been proposed, particularly when bradycardia recurs upon re-exposure after prior tolerance [8]. This hypothesis is supported by reports of bradycardia emerging during subsequent cytarabine cycles but not during earlier ones, suggesting sensitization or immunologic reactivity. In our patient, possible explanations include immune-mediated hypersensitivity, transient myocarditis, or cumulative subclinical toxicity involving both myocardial and conduction tissue, potentially amplified by prior doxorubicin exposure. The recurrence across varying dosage levels further supports an idiosyncratic or immune-mediated rather than purely dose-dependent mechanism.

Several risk factors are known to increase susceptibility to chemotherapy-related cardiotoxicity, including high cumulative or daily doses, rapid infusion rates, concurrent cardiotoxic drugs (particularly anthracyclines), pre-existing cardiovascular disease, electrolyte abnormalities (hypokalemia, hypomagnesemia), female sex, advanced age, and prior mediastinal radiation [12]. However, our patient had no baseline cardiac comorbidities, normal electrolytes, and preserved cardiac function before therapy, suggesting that cytarabine itself was the primary driver of her recurrent bradycardia, with concurrent exposure to doxorubicin potentially contributing to transient LV dysfunction.

In clinical practice, most cytarabine-associated bradycardia episodes are self-limiting, are often asymptomatic, and rarely require specific intervention [8, 12]. Management involves excluding reversible causes such as thyroid dysfunction, electrolyte imbalances, and infections, alongside ECG monitoring and supportive care. In rare cases where bradycardia is severe or symptomatic, temporary discontinuation of cytarabine or pacing support may be necessary.

The likelihood that cytarabine caused bradycardia in this case was assessed using the Naranjo Adverse Drug Reaction Probability Scale, yielding a score of 9, consistent with a definite adverse drug reaction [15]. This conclusion was supported by the clear temporal relationship between cytarabine administration and onset of bradycardia, resolution after drug discontinuation, recurrence upon rechallenge, absence of alternative explanations, and objective documentation of heart rate decline during monitoring.

A key feature of this case is the recurrence of bradycardia upon rechallenge with cytarabine. While previous reports have described both successful and unsuccessful rechallenges, such data remain limited. Recurrence strengthens the causal association and supports a definite adverse drug reaction according to the Naranjo scale [15]. This finding has important clinical implications, as cytarabine remains a cornerstone of AML therapy, and decisions regarding continuation or substitution directly affect disease control.

Our patient’s course highlights several important clinical lessons. First, cytarabine can induce clinically significant bradycardia and may lead to a transient left ventricular dysfunction even in the absence of pre-existing cardiac disease or concurrent atrioventricular (AV)-nodal-blocking agents. Second, recurrence after rechallenge underscores the need for vigilance and suggests that permanent discontinuation should be considered for severe or symptomatic cases. Third, continuous cardiac monitoring during cytarabine infusion facilitates early detection and timely management of conduction abnormalities, especially in patients receiving intermediate- to high-dose therapy. Fourth, concurrent exposure to anthracyclines such as doxorubicin may amplify transient myocardial effects, emphasizing the importance of considering cumulative cardiotoxic risk in multi-agent chemotherapy regimens.

From a broader perspective, this case adds to the limited but growing body of literature on cytarabine-associated arrhythmias. By documenting both delayed onset and recurrence after rechallenge, as well as transient LV systolic impairment, it reinforces the potential spectrum of cytarabine-induced cardiotoxicity and emphasizes the need for individualized risk–benefit evaluation when considering continued exposure. Larger pharmacovigilance studies are warranted to better define the true incidence, predisposing factors, and pathophysiologic mechanisms of this rare but clinically relevant adverse event.

## Conclusion

Cytarabine-induced bradycardia is a rare and underrecognized complication of AML therapy. This case illustrates that bradycardia can occur even in patients without traditional cardiotoxic risk factors, may present with delayed onset, and may recur upon rechallenge. Transient reductions in left ventricular function may accompany rhythm abnormalities, potentially influenced by prior anthracycline exposure, suggesting a broader cardiotoxic spectrum. Awareness of this phenomenon is essential to avoid unnecessary treatment discontinuation and to ensure patients complete potentially curative therapy under careful cardiac monitoring. Clinicians should maintain a high index of suspicion for cytarabine-induced bradyarrhythmias and incorporate close cardiac surveillance into standard care for patients receiving this agent.

## Strengths and limitations

### Strengths

This report provides a detailed and rare description of cytarabine-induced recurrent sinus bradycardia with transient left ventricular dysfunction in a patient with acute myeloid leukemia. The clear temporal association between cytarabine administration and onset of bradycardia, resolution after discontinuation, and recurrence upon rechallenge strongly supports a causal relationship. Comprehensive clinical assessment and exclusion of common reversible causes such as electrolyte imbalance, thyroid dysfunction, and infection strengthen diagnostic confidence. Moreover, this case illustrates an unusual delayed-onset pattern and highlights that a transient reduction in left ventricular ejection fraction may, in part, reflect underestimation of true systolic function due to bradycardia. The inclusion of a focused literature review enhances its scientific and educational contribution by contextualizing this event within the broader landscape of chemotherapy-related cardiotoxicity.

### Limitations

Several limitations should be acknowledged. First, as a single-patient observation, the findings cannot be generalized, and definitive causality cannot be established despite supportive evidence. Second, a baseline ECG before chemotherapy initiation and during the first episode of bradycardia was not available, which limits the ability to assess pre-existing conduction abnormalities and precludes confirmation of the rhythm disturbance and comparison with subsequent events, respectively. Third, resource constraints prevented cytogenetic studies and detailed flow cytometry, which could have provided additional diagnostic and prognostic information regarding leukemia subtype and risk profile. Continuous cardiac monitoring was available only during chemotherapy administration, possibly underestimating transient or asymptomatic arrhythmias. Advanced cardiac imaging, such as strain analysis or cardiac MRI, was not performed due to similar limitations. Finally, the scarcity of published data on cytarabine-associated bradyarrhythmias restricts broader mechanistic interpretation and external comparison.

## Data Availability

The datasets used and/or analyzed during the current study are available from the corresponding author on reasonable request.

## Abbreviations

AML: Acute myeloid leukemia
LVEF: Left ventricular ejection fraction
IDAC: Intermediate-dose cytarabine
ECG: Electrocardiogram/electrocardiography
LV: Left ventricle
EF: Ejection fraction
CRP: C-reactive protein
CT: Computed tomography
MRI: Magnetic resonance imaging
